# Parental Autonomy Granting and School Functioning among Chinese Adolescents: The Moderating Role of Adolescents’ Cultural Values

**DOI:** 10.3389/fpsyg.2017.02161

**Published:** 2017-12-13

**Authors:** Cixin Wang, Kieu Anh Do, Leiping Bao, Yan R. Xia, Chaorong Wu

**Affiliations:** ^1^Counseling, Higher Education, and Special Education, University of Maryland, College Park, College Park, MD, United States; ^2^Asian American Studies, University of Maryland, College Park, College Park, MD, United States; ^3^Institute of Youth and Juvenile Studies, Shanghai Academy of Social Sciences, Shanghai, China; ^4^Child, Youth and Family Studies, University of Nebraska–Lincoln, Lincoln, NE, United States; ^5^Institute for Clinical and Translational Science, University of Iowa Health Care, Iowa City, IA, United States

**Keywords:** autonomy granting, independent self-construal, interdependent self-construal, familism, school adjustment, grades

## Abstract

School adjustment and achievement are important indicators of adolescents’ well-being; however, few studies have examined the risk and protective factors predicting students’ school adjustment and achievement at the individual, familial, and cultural level. The present study examined the influences of individual and familial factors and cultural values on Chinese adolescents’ school functioning (e.g., school adjustment and grades). It also tested whether cultural values moderated the relationship between parenting and adolescents’ school functioning. Self-report data were collected from a stratified random sample of 2,864 adolescents (51.5% female, mean age = 15.52 years, grade 6th – 12th) from 55 classrooms, in 13 schools in Shanghai, China. Results showed that self-esteem (*b*_se→adj_ = 0.05, *SE* = 0.01, *p* < 0.001; *b*_se→grades_ = 0.08, *SE* = 0.02, *p* < 0.001), parent–adolescent conflict (*b*_conflict→adj_ = -0.03, *SE* = 0.00, *p* < 0.001; *b*_conflict→grades_ = -0.04, *SE* = 0.01, *p* < 0.001), and conformity to parental expectations (*b*_conform→adj_ = -0.03, *SE* = 0.02, *p* < 0.05; *b*_conform→grades_ = 0.10, *SE* = 0.04, *p* < 0.05) all had significant effects on both school adjustment and grades, respectively. More importantly, results showed that independent self-construal moderated the relationship between parental autonomy granting and adolescents’ grades (*b*_indepxautom_ = 0.06, *SE* = 0.02, *p* < 0.01). The findings suggest that cultural values may influence adolescents’ appraisal of parental autonomy granting, which then impacts their school functioning.

## Introduction

School adjustment and achievement are important indicators of adolescents’ well-being. Most research on adolescents’ school functioning tends to use samples collected in Western cultures. With increased globalization, it is critical to extend this research to include adolescents in non-Western contexts, such as China. For example, there are on-going debates regarding the beneficial effects of parental autonomy support on adolescents’ outcomes, which is evident in Western cultures where independence is greatly valued. However, we have limited information about whether it is also beneficial in East Asian cultures, where interdependence and connectedness are emphasized (Bao and Lam, 2008; Qin et al., 2009). Self-determination theory (SDT) posits that the need for personal autonomy is universal, and parenting practices that support adolescent autonomy tend to produce positive youth outcomes including better school functioning (Grolnick et al., 1997; Deci and Ryan, 2000). However, cultural relativists have challenged these universal assumptions, arguing that cultural background impacts how adolescents appraise certain parenting practices (Soenens et al., 2015). To add new understanding to this debate, the current study examined the influences of individual and familial factors on Chinese students’ school functioning. It also tested cultural values as moderators on the relationship between parental autonomy granting and adolescents’ school functioning.

### Parenting and School Functioning

According to Confucian philosophy, education is the means for upward social mobility for both the individual and his or her family. Therefore, Chinese parents are highly involved in the lives of their adolescent children, paying special attention to their school functioning. Chinese parents also tend to be more restrictive in their parenting practices than North American parents (Kelley and Tseng, 1992; Chao, 1994) and use more psychologically controlling strategies (instead of autonomy supportive strategies; Cheung and Pomerantz, 2011). Additionally, Chinese parents are more likely to encourage children’s connectedness and less likely to encourage autonomy (Liu et al., 2005) than their North American counterparts. Chinese youth, influenced by interdependent cultural values, are less likely to view adolescence as a time of increased independence and individuation from parents (Qu et al., 2016). They also tend to anticipate gaining autonomy at an older age compared to American adolescents (Feldman and Rosenthal, 1991).

From the perspective of SDT, since the need for personal autonomy is universal (Deci and Ryan, 2000), parental autonomy support is posited to function similarly in both collectivist and individualist cultures (Marbell and Grolnick, 2013). “Even in interdependent society, when parents take children’s perspectives, allow them to voice their opinions, and provide choices, it is associated with positive outcomes” (Marbell and Grolnick, 2013, p. 89). Several cross-cultural studies have supported this view, showing that higher autonomy support predicted better psychological and academic functioning for students in the United States, as well as in collectivist Asian cultures, including China (Hasebe et al., 2004; Vansteenkiste et al., 2005; Supple et al., 2009).

However, cultural relativists maintained that certain parenting strategies may function differently in different cultural settings. Consistent with this perspective, research has shown that Chinese, Chinese American, and Asian American adolescents interpret parental control differently than their Caucasian peers. Instead of perceiving control as a violation of their individualism and privacy, they tend to view it as an expression of love and care (Lam, 2003; Cheung and McBride-Chang, 2008). Similarly, some adolescents in collectivist cultures (e.g., Ghana) regarded certain autonomy granting questionnaire items negatively, interpreting them as lack of parental support (Marbell and Grolnick, 2013). Therefore, parental autonomy granting and control may have different meanings and functions in Chinese society, where interdependence and collectivism are valued (Bao and Lam, 2008).

### Parental Autonomy Granting

Additionally, non-significant relationships between autonomy granting and school functioning have also been documented among Chinese adolescents (Cheung and Pomerantz, 2011; Cheung et al., 2016), suggesting that there may be potential moderating effects that have not been investigated. Theoretically, cultural values and familial factors may interact to predict youth outcomes (Bronfenbrenner and Morris, 2006). In other words, adolescents with different levels of cultural values may benefit from different types of parenting styles. Considering the large within-group variation on cultural values, it is possible that these intracultural variations may moderate the relationship between parental autonomy granting and adolescents’ school functioning. Based on our knowledge, no published studies have examined this moderation effect among Chinese youth. The current study seeks to advance current understanding of the role of parental autonomy granting on school functioning by examining the moderating effect of cultural values.

### Conformity to Parental Expectations

Conformity to parental expectations is another concept related to autonomy granting. For example, Chinese adolescents showed more willingness to adhere to their parents’ academic expectations compared to their American peers (Chen and Lan, 1998). Although some Western researchers expressed concerns that too much conformity might prevent adolescents from developing social competency and autonomy (Allen et al., 1989), Chinese adolescents who have high levels of conformity to their parents actually had higher school engagement and stronger school motivation (Shen, 2011). Additionally, parent–adolescent conflict is another important family variable that has been found to impact youth outcomes in China, including school adjustment difficulties. For example, Shek (1997, 2002) postulated that conflict with parents might act as a potential stressor affecting adolescents’ emotional well-being and impacting their school adjustment.

### Cultural Values

Ecological Systems Theory (Bronfenbrenner, 1992; Bronfenbrenner and Morris, 2006) proposes that behaviors are influenced by multiple systems (individual, family, school/classroom, culture) as well as their interactions. Few studies have explored how cultural values might impact school functioning, particularly at both the individual and classroom level. One way to measure cultural values is on the continuum of independence and interdependence. According to self-construal theory (Markus and Kitayama, 1991), there are differences in the cultural orientation of the self between people in the East (e.g., China and Japan) and those in the West (e.g., United States and Canada). Individuals in the East tend to have higher interdependent self-construal, emphasizing their relationship to the social group and considering the attainment of group goals and harmony to be of importance. On the other hand, people in the West tend to have higher independent self-construal, with a strong focus on the individual’s identity and uniqueness, emphasizing the pursuit of personal goals and achievements (Cross, 1995; Singelis et al., 1999). In general, collectivism and interdependence are valued in Chinese society and have been linked to positive youth outcomes, such as lower aggression (Li et al., 2010).

### Familism

Additionally, Chinese society also values a strong sense of familism. Few studies have investigated the effects of familism on students’ educational outcomes in China or how this cultural value might interact with parenting to predict adolescents’ school functioning. Research in the United States has found that Asian American youth, including those of Chinese descent, have a stronger sense of family obligation (one type of familism) than European American youth (Tseng, 2004). Furthermore, higher emphasis on family obligation was linked to stronger academic motivation, stronger educational aspirations, and more time studying among Asian American adolescents (Fuligni et al., 1999; Tseng, 2004). There are also considerable intracultural variations among adolescents, which deserve attention from researchers in order to understand individual differences in parenting and school functioning within a given context. Based on our knowledge, no published studies have examined the relationship between intracultural variations in cultural values and school functioning among Chinese adolescents.

Furthermore, no studies have examined classroom-level cultural values as predictors of adolescents’ school functioning in China. Different from their American counterparts, Chinese adolescents typically spend their school days with the same group of peers in the same classroom (instead of switching classrooms for different subjects). Consequently, a significant amount of socialization occurs in the classroom, resulting in unique classroom behavioral norms and values. Tracking students (assigning students into different classrooms) based on their grades is also a common practice in Chinese schools, potentially leading to different classroom norms even within the same school. Our study will advance previous research by examining the effects of cultural values and their interaction with parenting at both the individual and classroom level on adolescents’ school functioning.

### Individual-Level Factors Influencing School Functioning

Various students’ characteristics, such as gender, age, only-child status and self-esteem, also have been found to contribute to school outcomes. Some researchers showed that Chinese girls tend to have better academic achievement than boys (Shen, 2011; Liu et al., 2014), while others did not find these gender differences (Schwartz et al., 2013). Moreover, older Chinese students tend to have lower GPAs (grade point averages) compared to younger students (Shen, 2011), and self-esteem has been found to positively relate to school motivation and grades (Tsai et al., 2001; Shen, 2011).

Due to the one-child policy, most adolescents in China are the only-child at home. Parents place high expectations on their adolescent to succeed academically. This has presented a unique phenomenon for adolescent development. It is possible that Chinese parents have increased financial means and resources to support their only-child’s academic achievement compared to parents who have multiple children. Findings relating to the only-child status are less consistent. For instance, Chen et al. (1994) found that only-children and children with siblings did not differ significantly on normative school behaviors (moral, intellectual, physical areas), scholastic excellence, and academic achievement (in Chinese language and math) in elementary school. In a more recent study, Zhou et al. (2016) found that Chinese only-children have similar math achievement scores compared to their peers with siblings; however, they scored lower on other cognitive measures, such as processing speed and working memory. Due to these inconsistent findings, we will examine the role of individual variables on school functioning in the Chinese context.

### Current Study

The current study was guided by the SDT framework (Grolnick et al., 1997; Deci and Ryan, 2000) and ecological systems theory (Bronfenbrenner and Morris, 2006). Using multilevel modeling, the goal of the current study was to examine the influence of individual (e.g., demographic characteristics, self-esteem), familial (e.g., parental autonomy granting, conformity to parental expectations, parent–adolescent conflict), and cultural factors (e.g., familism, interdependence, independence) on Chinese students’ school functioning (e.g., school adjustment and grades). The second goal was to examine how cultural values moderate the relationship between parental autonomy granting and adolescents’ school functioning. Specifically, the study addressed two main research questions:

(1) Do individual and familial factors as well as intracultural variations in cultural values (individual and classroom level) influence students’ school functioning? *Hypothesis 1:* We hypothesized that self-esteem and being the only-child would predict better school functioning. *Hypothesis 2:* For familial variables, we expected that higher levels of conformity to parental expectations would predict better school functioning; while higher levels of parent–adolescent conflict would predict worse school functioning. *Hypothesis 3:* Considering the collectivistic and interdependent cultural norms in China, students who value interdependence and familism and those who come from classrooms that reinforced these values would have better school functioning.
(2) Do intracultural variations in cultural values (both the individual and classroom level) moderate the relationship between parental autonomy granting and adolescents’ school functioning? *Hypothesis 4:* We expected parental autonomy granting to have a positive effect on Chinese adolescents’ school functioning and that cultural values would moderate this relationship.

## Materials and Methods

### Participants and Procedure

To obtain a representative sample, we collected data from a stratified random sample of 2,864 students (6th through 12th grade) from 55 classrooms, in 13 schools in Shanghai, China in 2007. The average age of the sample was 15.52 years (*SD* = 1.62), with the majority being female (51.5%) and the only-child in their family (86.1%). The ethical review committee at Shanghai Academy of Social Sciences approved this study. The passive consent method was used; whereby, parents were notified about the research and were given the opportunity to withdraw their children from study participation. Adolescents then gave assent to participate in this study. Students completed the survey at school, containing several measures and a demographic questionnaire (age, gender, grade, only-child status). No identifying information was collected.

### Measures

We collected data using the following measures to answer our research questions related to how cultural values (independence, interdependence, and familism), as well as other individual (self-esteem, only-child status) and familial factors (parental autonomy granting, conformity to parental expectations, parent–adolescent conflict) relate to students’ school functioning.

#### Self-Esteem

Self-esteem was measured using the Youth Sources of Self-Esteem Inventory, a revised version of the Adult Sources of Self-Esteem Inventory (ASSEI; Watkins et al., 1998, 2000). The inventory has two subscales: (1) satisfaction subscale (measures self-esteem) and (2) importance subscale (measures self-construal). The unweighted satisfaction subscale was used to assess self-esteem in this study. The measure contains 20 items, asking participants to rate their level of satisfaction on 20 different attributes, such as accomplishments, clothing/grooming, physical attractiveness, and spirituality. Ratings ranged from 0 (*not satisfied*) to 10 (*extremely satisfied*). The subscale has shown high internal consistency (Seegan et al., 2012) and good test–retest reliability. It has also shown good construct validity with significant positive correlations with the Rosenberg’s Self-Esteem Scale (*r* = 0.37 and 0.52; see Watkins et al., 1998) and significant negative correlations (*r* = -0.14 and -0.37) with Eysenck’s Neuroticism Scale (see Watkins et al., 2000). In this study, Cronbach’s α was 0.94.

#### Parental Autonomy Granting

Parental autonomy granting is the extent to which adolescents perceived their mothers (10 items) and fathers (10 items) allowing them to make decisions regarding choices about friendships, educational goals, career plans, and life-style preferences (e.g., “*Father/Mother gives me enough freedom*”). The items came from the Parent Behavior Measure (Bush et al., 2002). Ratings were based on a four-point Likert scale, ranging from 1 (*strongly disagree*) to 4 (s*trongly agree*). Participants’ responses were averaged, with higher scores indicating higher levels of autonomy granting. The measure has been validated with a Chinese sample using confirmatory factor analysis (CFA) showing good structural validity with factor loadings ranging from 0.36 to 0.69 (Supple et al., 2009). Fit indices indicated good model fit: CFI_mothers_ = 0.91, CFI_fathers_ = 0.93, RMSEA_mothers_ = 0.06, RMSEA_fathers_ = 0.06 (Supple et al., 2009). Reliabilities were also adequate, ranging from 0.77 to 0.83 (Shen, 2011). In this study, reliability for adolescents’ perception of father’s (α = 0.90) and mother’s autonomy granting (α = 0.90) were high, with significantly high correlations between paternal and maternal scores. Preliminary analyses with separate maternal and paternal autonomy granting scores showed similar results with all outcomes of interest. Although this is not the foci of the current study, these preliminary results suggest this parent–child dynamic function similarly for mothers and fathers; therefore, the two scores were combined to create an overall parental score. The combined Cronbach’s α-value was 0.90.

#### Parent–Adolescent Conflict

Two items were used to measure the frequency and intensity of parent–adolescent conflict (Bush et al., 2013). The first item asked adolescents to rate how often they argue with their parents on a five-point Likert scale, from 1 (*almost never*) to 5 (*several times a day*). The second item asked adolescents to rate the severity of their arguments with their parents, from 1 (*very minor if happens*) to 5 (*always very severe*). Bush et al. (2013) showed that the items have adequate reliabilities (α = 0.70–0.76), assessing a single construct. The researchers further found negative associations between fathers’ parental support and father–adolescent conflict for both boys (*b* = -0.14, *p* < 0.05) and girls (*b* = -0.13, *p* < 0.05), showing divergent validity. On the other hand, maternal punitiveness was related to more mother–adolescent conflicts for both boys (*b* = 0.32, *p* < 0.001) and girls (*b* = 0.28, *p* < 0.001), showing good convergent validity. In this study, the two items were multiplied together to form an indicator of parent–adolescent conflict. Higher scores indicate higher levels of conflict.

#### Conformity to Parental Expectations

Conformity to parental expectations was measured using the Conformity to Parental Expectations Scale (Peterson et al., 1985, 1999). It consists of items assessing the degree to which adolescents adhere to their parents’ (nine items about their mothers’, nine items about their fathers’) beliefs, values and expectations relating to dating, education, friends, careers, and leisure activities. For example, “*If Mother/Father wanted me to choose a particular career, then I would try to prepare for that career*.” Ratings varied from 1 (*strongly disagree*) to 4 (*strongly agree*). Responses were averaged, with higher scores indicating higher levels of conformity. Peterson et al. (1985) found that this scale captured one single conformity factor with adequate internal reliability (0.74). In addition, mothers’ and fathers’ support positively correlated with higher conformity (*r* = 0.16 and 0.14, *p*s < 0.05), suggesting good construct validity. This measure has also been used in cross-cultural studies, showing alphas ranging from 0.77 to 0.85 (Bush et al., 2013). In this study, reliabilities were high for adolescents’ conformity to their fathers’ (α = 0.89) and their mothers’ expectations (α = 0.89). Preliminary analyses with separate maternal and paternal scores showed similar results with the outcomes of interest; therefore, both were combined into one conformity to parental expectation score (α = 0.89).

#### Cultural Self-Construal

Adolescents’ independent and interdependent self-construal were assessed using the importance subscale of the Youth Sources of Self-Esteem Inventory (Watkins et al., 1998, 2000). Participants rated the importance of 20 attributes on a scale from 0 (*not important*) to 10 (*extremely important*). Sample items included “*Relationships with my family are important to me*” (interdependent self-construal), and “*Doing what I set out to do personally and meeting the goals I set for myself is important to me*” (independent self-construal). This measure has also been validated across more than 20 different countries including China. Comparison of the factor loadings within and between countries supported a two-factor solution with good model fit for both the independence (Tucker’s ϕ = 0.74–0.99) and interdependence subscale (Tucker’s ϕ = 0.70–0.98), suggesting good structural validity (Van de Vijver and Watkins, 2006). The instrument also showed good internal reliabilities, with alpha values greater than 0.80 for both interdependent and independent self-construal (Watkins et al., 2000; Pekerti and Kwantes, 2011). In this study, Cronbach’s alphas for interdependent self-construal (α = 0.90) and independent self-construal (α = 0.87) were also high.

#### Familism

Five items from Bardis’ (1959a) Familism Scale were modified and used to measure familism in this study. Participants rated their loyalties, obligations, and feelings toward their family (e.g., “*A person should be completely loyal to his family*”). Responses ranged from 1 (*strongly disagree*) to 4 (*strongly agree*). The original items showed good discriminant validity in distinguishing between a sample of youth in Greece (*M* = 46.75) and three samples of students in the United States (Mennonite college students *M* = 29.04, high school Methodist students *M* = 31.32, and college Methodist students *M* = 24.41; Bardis, 1959b). Specifically, the Greek sample displayed higher familism than all three United States samples, while the three United States samples showed similar levels of familism to each other. The scale also showed high test–retest reliability (α = 0.90; Bardis, 1959a) as well as high internal consistency with a sample of Korean and Korean American caregivers (α = 0.83; Youn et al., 1999). In this study, the instrument also showed adequate reliability (α = 0.75). Items were averaged, with higher scores indicating higher levels of familism.

#### School Adjustment

The Perceived Negative Adjustment to School Scale (Xia et al., 2015; Wang et al., 2016), a modified version of the Denver Youth Survey Interview Schedule (Elliot, 1990), was used to assess students’ levels of perceived school adjustment. This eight-item instrument measured students’ academic engagement (e.g*., “I complete all my homework”)*, school belonging (e.g., “*I don’t feel as if I really belong in school”)*, and school social functioning (e.g., “*I am often mad at other students at school*”). Responses were based on a five-point Likert scale, ranging from 1 (*strongly disagree*) to 5 (*strongly agree*). Five items with negative wordings were reverse coded. An average school adjustment score was calculated, with higher scores indicating better school adjustment. In a recent study with adolescents in China, school adjustment difficulty was significantly correlated with lower self-esteem (*r* = -0.29, *p* < 0.001) but was positively correlated with more depressive symptoms (*r* = 0.22, *p* < 0.001) and more problem behaviors (*r* = 0.42, *p* < 0.001), suggesting good construct validity (Wang et al., 2016). In this study, the overall scale also demonstrated high reliability (α = 0.85).

#### Grade Rank

Students reported their class rank on their last exam. Ranking ranged from 1 (*top 5th of the class*), 2 (*between 6th and 10th place*), 3 (*between 11th and 20th place*), 4 (*between 21st and 30th place*), and 5 (*30th or higher*). Ranks were reversed coded, with higher scores indicating better ranking in the class. Grade ranks were used in this study because posting the class ranking in public is a common practice in Chinese schools. Using grade rank instead of actual grades may reduce the variations in grading due to different tests and grading procedures by teachers.

### Data Analysis

Multilevel modeling was performed using SAS PROC GLIMMIX, due to the nested nature of the data, whereby students were nested within classrooms, classrooms nested within schools, and schools nested within school districts. Three random intercepts were included, with the assumption that there were variations at the classroom, school, and district level when covariates were controlled. All variables used in the analysis, except the three cultural values (independent and interdependent self-construal and familism), were centered at their grand means. For each cultural value, two separate variables were created to represent the within-classroom (referred to as individual-level) and between-classroom (referred to as classroom-level) effects. The within-classroom predictors were centered at the classroom mean. For instance, within-classroom independent self-construal for a student was derived from subtracting the classroom’s average score from his/her individual score. The between-classroom predictors were calculated using the average score of the students in the classroom, which were centered at their grand mean to indicate the average differences between classrooms. To examine the interaction effect, six interaction terms between parental autonomy granting and individual-level and classroom-level cultural values were created.

## Results

### Descriptive Statistics

**Table 1** shows the descriptive statistics and **Table 2** displays the correlations between the variables. There were significant correlations between school adjustment and all other variables, except conformity to parental expectations. Grade rank was significantly correlated with self-esteem, parental autonomy granting, and parent–adolescent conflict. Cultural values (independence, interdependence, and familism) positively correlated with each other and with parental autonomy granting.

**Table 1:** Mean and standard deviation of variables of interest by gender and grade level.

	Total	Male	Female	6th	7th	8th
	Mean (*SD*)	Mean (*SD*)	Mean (*SD*)	Mean (*SD*)	Mean (*SD*)	Mean (*SD*)
Self-esteem	7.25 (1.90)	7.16 (2.00)	7.33 (1.80)	8.12 (1.25)	8.15 (1.69)	9.43 (0.81)
Parental autonomy granting	3.14 (0.64)	3.10 (0.69)	3.17 (0.59)	3.10 (0.40)	3.19 (0.71)	3.55 (0.64)
Parent–adolescent conflict	2.96 (2.65)	2.89 (2.91)	3.03 (2.39)	2.23 (1.96)	2.46 (1.91)	4.50 (4.95)
Conformity to parents	2.33 (0.73)	2.39 (0.77)	2.27 (0.68)	2.62 (0.46)	2.40 (0.77)	3.50 (0.71)
Interdependent self-construal	8.29 (1.70)	8.08 (1.84)	8.48 (1.54)	8.43 (1.09)	8.83 (0.93)	9.55 (0.64)
Independent self-construal	7.64 (1.70)	7.45 (1.82)	7.83 (1.57)	7.26 (1.52)	8.14 (1.19)	8.65 (0.64)
Familism	2.79 (0.13)	2.78 (0.14)	2.79 (0.12)	2.95 (0.04)	2.82 (0.13)	2.66 (0.00)
School adjustment	3.68 (0.55)	3.63 (0.58)	3.72 (0.50)	4.15 (0.47)	4.04 (0.51)	3.94 (0.09)
Grade rank	1.75 (1.27)	1.58 (1.26)	1.92 (1.25)	1.38 (0.77)	1.97 (1.24)	2.40 (0.71)


**SD*, standard deviation, *N* = 2,864.*

**Table 2:** Bivariate correlations between all variables.

	1	2	3	4	5		6	7	8	9	10	11	12
1. Self-esteem	–												
2. Autonomy granting	0.22^∗∗∗^	–											
3. Parent–adolescent conflict	-0.18^∗∗∗^	-0.21^∗∗∗^	–										
4. Conformity to parents	0.06^∗∗^	0.24^∗∗∗^	-0.14^∗∗∗^	–									
5. IL_Interdependent	0.53^∗∗∗^	0.17^∗∗∗^	-0.12^∗∗∗^	0.01	–								
6. CL_Interdependent	0.36^∗∗∗^	0.05^∗^	-0.03	-0.04	0.00		–						
7. IL_Independent	0.48^∗∗∗^	0.11^∗∗∗^	-0.01	0.02	0.77^∗∗^		0.00	–					
8. CL_Independent	0.34^∗∗∗^	0.03	-0.00	-0.03	0.00		0.95^∗∗∗^	0.00	–				
9. IL_Familism	0.11^∗∗∗^	0.16^∗∗∗^	-0.15^∗∗∗^	0.17^∗∗∗^	0.16^∗∗∗^		0.00	0.12^∗∗∗^	0.00	–			
10. CL_Familism	0.11^∗∗∗^	0.08^∗∗∗^	-0.05^∗^	0.02	0.00		0.28^∗∗∗^	0.00	0.22^∗∗∗^	0.00	–		
11. School adjustment	0.25^∗∗∗^	0.16^∗∗∗^	-0.23^∗∗∗^	0.01	0.17^∗∗∗^		0.13^∗∗∗^	0.10^∗∗∗^	0.11^∗∗∗^	0.10^∗∗∗^	0.07^∗∗∗^	–	
12. Grade rank	0.11^∗∗∗^	0.06^∗∗^	-0.10^∗∗∗^	0.00	0.03		0.01	0.02	0.01	0.00	-0.02	0.12^∗∗∗^	–


*IL, Individual-level; CL, class-level. ^∗^*p* < 0.05; ^∗∗^*p* < 0.01; ^∗∗∗^*p* < 0.001.*

### Associations between the Main Study Variables

Results showed that male students had significantly lower school adjustment (*b* = -0.06, *SE* = 0.02, *p* < 0.01) and had worse grade ranking (*b* = -0.36, *SE* = 0.05, *p* < 0.001) than female students. Only-child status positively predicted better grade ranking (*b* = 0.16, *SE* = 0.07, *p* < 0.05) but not school adjustment (*b* = 0.03, *SE* = 0.03, *p* > 0.05). Self-esteem significantly predicted both students’ school adjustment (*b* = 0.04, *SE* = 0.01, *p* < 0.001) and their grade ranking (*b* = 0.08, *SE* = 0.02, *p* < 0.001).

For the familial variables, parental autonomy granting (*b* = 0.06, *SE* = 0.02, *p* < 0.001) significantly predicted students’ school adjustment but did not predict grade ranking. Parent–adolescent conflict negatively predicted both students’ school adjustment (*b* = -0.03, *SE* = 0.00, *p* < 0.001) and grade ranking (*b* = -0.04, *SE* = 0.01, *p* < 0.001). Contrary to our hypothesis, conformity to parental expectations (*b* = -0.09, *SE* = 0.04, *p* < 0.01) negatively predicted grade ranking. Students who had higher levels of conformity to their parents reported worse grades. For cultural values, individual-level interdependent self-construal (*b* = 0.02, *SE* = 0.01, *p* < 0.001) and individual-level familism (*b* = 0.05, *SE* = 0.02, *p* < 0.01) significantly predicted students’ school adjustment.

### Interaction Effects between Parental Autonomy Granting and Cultural Values

**FIGURE 1:**
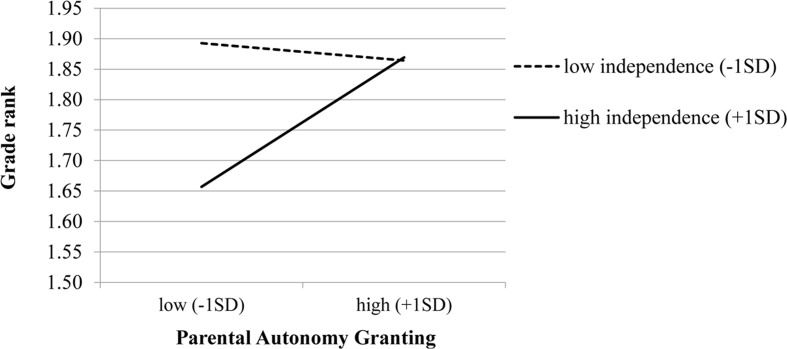
Interaction between parental autonomy granting and individual-level independent self-construal. Higher grade rank means better grades. Lower independence = 1 *SD* below the mean, and high independence = 1 *SD* above the mean when all other variables are at the mean.

Due to the high correlation between independent and interdependent self-construal, we examined the moderation models separately for each predictor. Results partially supported our hypothesis concerning the interaction effect, showing that parental autonomy granting interacted with individual-level independent self-construal to predict grade rank (*b* = 0.06, *SE* = 0.02, *p* < 0.01; **Tables 3**, **4**). As illustrated in **Figure 1**, for students who reported higher independent self-construal (1 *SD* above the mean), more parental autonomy granting resulted in better grade ranking. On the other hand, for students who reported lower independent self-construal (1 *SD* below the mean), more autonomy granting resulted in lower grade ranking. None of the other interactions were significant.

**Table 3:** Multilevel model predicting school adjustment and grade ranking: interdependent self-construal.

	School adjustment	Grade rank
		
	Model	Model
	*b* (*SE*)	*b* (*SE*)
Intercept	3.68 (0.06)^∗∗∗^	1.82 (0.13)^∗∗∗^
Age	-0.03 (0.01)^†^	-0.01 (0.03)
Male	-0.06 (0.02)^∗∗^	-0.36 (0.05)^∗∗∗^
Only child	0.03 (0.03)	0.16 (0.07)^∗^
Self-esteem	0.04 (0.01)^∗∗∗^	0.08 (0.02)^∗∗∗^
Parental autonomy granting	0.06 (0.02)^∗∗∗^	0.08 (0.04)^†^
Parent–adolescent conflict	-0.03 (0.00)^∗∗∗^	-0.04 (0.01)^∗∗∗^
Conformity to parents	-0.03 (0.02)^†^	-0.09 (0.04)^∗^
CL_Interdependent self-construal	0.02 (0.02)	-0.01 (0.05)
IL_Interdependent self-construal	0.02 (0.01)^∗∗^	-0.04 (0.02)^†^
CL_Familism	0.07 (0.11)	-0.14 (0.23)
IL_Familism	0.05 (0.02)^∗∗^	0.01 (0.05)
CL_Interdependent × Autonomy	0.03 (0.02)	0.05 (0.05)
IL_Interdependent × Autonomy	0.02 (0.01)^†^	0.01 (0.02)
CL_Familism × Autonomy	-0.19 (0.13)	-0.16 (0.32)
IL_Familism × Autonomy	0.03 (0.02)	0.04 (0.05)
AIC	3,390.71	7,496.16
BIC	3,389.15	7,498.60
-2LL	3,382.71	7,488.16


*CL, class-level; IL, individual-level; *N* = 2,864. ^†^*p* < 0.10; ^∗^*p* < 0.05; ^∗∗^*p* < 0.01; ^∗∗∗^*p* < 0.001.*

**Table 4:** Multilevel model predicting school adjustment and grade ranking: independent self-construal.

	School adjustment	Grade rank
		
	Model	Model
	*b* (*SE*)	*b* (*SE*)
Intercept	3.67 (0.06)^∗∗∗^	1.82 (0.13)^∗∗∗^
Age	-0.03 (0.01)^†^	-0.01 (0.03)
Male	-0.06 (0.02)^∗∗^	-0.36 (0.05)^∗∗∗^
Only child	0.03 (0.03)	0.16 (0.07)^∗^
Self-esteem	0.05 (0.01)^∗∗∗^	0.08 (0.02)^∗∗∗^
Parental autonomy granting	0.06 (0.02)^∗∗∗^	0.07 (0.04)
Parent–adolescent conflict	-0.03 (0.00)^∗∗∗^	-0.04 (0.01)^∗∗∗^
Conformity to parents	-0.03 (0.02)^∗^	-0.10 (0.04)^∗^
CL_Independent self-construal	0.00 (0.02)	-0.03 (0.05)
IL_Independent self-construal	0.01 (0.01)	-0.04 (0.02)^†^
CL_Familism	0.08 (0.11)	-0.12 (0.22)
IL_Familism	0.06 (0.02)^∗∗^	0.00 (0.04)
CL_Independent × Autonomy	0.02 (0.02)	0.05 (0.05)
IL_Independent × Autonomy	0.01 (0.01)	0.06 (0.02)^∗^
CL_Familism × Autonomy	-0.19 (0.13)	-0.15 (0.31)
IL_Familism × Autonomy	0.03 (0.02)	0.02 (0.05)
AIC	3,402.03	7,489.25
BIC	3,400.46	7,487.69
-2LL	3,394.03	7,481.25


*CL, class-level; IL, individual-level; *N* = 2,864. ^†^*p* < 0.10; ^∗^*p* < 0.05; ^∗∗^*p* < 0.01; ^∗∗∗^*p* < 0.001.*

## Discussion

The aim of this study was to examine individual, familial, and cultural factors on Chinese students’ school functioning, with a special focus on the interactions between reported parenting and cultural values. The results supported most of our hypotheses. Specifically, independent cultural values moderated the relationship between parental autonomy granting and grade rank. Adolescents who reported higher independent values are more likely to benefit from parenting behavior that provides greater autonomy. On the other hand, for adolescents who reported lower levels of independent values, parental autonomy granting predicted lower grade rank.

Although SDT guided this study on autonomy granting, we also share Soenens et al.’s (2015, p. 45) position of universalism without uniformity in that “psychological processes have both universal and context-specific features.” Cultural background may impact how autonomy granting is perceived by adolescents, and therefore parenting practices may have differential impacts on adolescents’ outcomes in different cultures (Greenfield et al., 2003; Soenens et al., 2015). However, which specific cultural variable may serve as a moderator has not been explicitly tested. Some cross-cultural studies failed to show country as a moderator on the relationship between autonomy granting and youth outcomes (Ferguson et al., 2011; Cheung et al., 2016), possibly due to the large within-culture variation in adolescents’ appraisal of parental autonomy support. Our finding expands current understandings relating to cultural values as moderating variables and helps to explain why parental autonomy granting may affect adolescents differently. It is also supported by Lamm et al. (2017), who found that Nso children from the collectivistic country of Cameroon demonstrated better delay-of-gratification performance than their peers from individualistic Germany. The authors concluded that the enhanced self-regulation of Nso children was due to parenting emphasis on hierarchical relational socialization rather than those that focus on promoting psychological autonomy. Therefore, psychological autonomy socialization goals may not always be superior depending on the cultural context.

Although cultural influences may exist, the divergent effects of parental autonomy support on youth’s outcomes in this current study and others could also be due to differences in measurement. Those that found universal effects may be using measures that are tapping into more global concepts of parental autonomy support, while studies such as ours are assessing more culture-specific domains. For instance, Griffith and Grolnick (2014) measured four different aspects of autonomy support and found that only acknowledgment and opinion exchange (but not parental allowance of choice and independent decision making) were significant predictors of child outcomes (e.g., school engagement) in Caribbean families. The autonomy granting measure we used was similar to the allowance of choice and independent decision-making subscales in Griffith and Grolnick’s (2014) study. Future works should consider employing different measures of autonomy support within the same study to tweeze out which component is more universal and which is more culture-specific.

Consistent with previous research (Marbell and Grolnick, 2013), we also found that parental autonomy granting significantly correlated with adolescents’ collectivist cultural values (interdependence and familism). This may seem counter-intuitive, but SDT also suggests that adolescents are more likely to internalize culture values if they are given the freedom to explore these values before making the decision to adhere to them. In other words, parental autonomy granting is not in conflict with interdependent cultural values (Marbell and Grolnick, 2013).

Consistent with our hypotheses and prior research, students who value interdependence and familism had better school adjustment. Our results also showed that independent self-construal significantly correlated with interdependent self-construal and familism. This finding is consistent with earlier arguments that value systems such as collectivism and individualism are not polar opposites (Li et al., 2010, p. 192). Individuals may endorse both value orientation in different contexts (Tamis-LeMonda et al., 2008). For example, Chinese adolescents may endorse more collectivist values in the peer context and more individualistic values in the learning context (Li et al., 2010).

Contrary to existing research (Zhou et al., 2016), we found that only-child status predicted better grades, possibly because families can afford more attention and resources to support one child in the home compared to those with multiple children. On the other hand, higher parent–adolescent conflict negatively predicted school functioning, which is consistent with the existing literature (Shek, 2002). Students who came from families with high levels of conflict did not seem to adjust well in school and had lower grade ranks. It is important to keep in mind that the reverse may also be possible. Students who perform poorly in school may be more likely to get into arguments/conflicts with their parents relating to their achievement.

Our study also yielded some unexpected findings that warrant further investigation. For instance, while conformity and grades were not correlated, it is unclear as to why stronger conformity to parental expectations predicted poorer grades after controlling for other individual and family variables. One possible explanation is that conformity to parental expectations may only be helpful to students who exhibit high levels of interdependent self-construal. More research is needed to explore these possible interactions. Second, contrary to our hypothesis, the moderation effect of independence was not significant for school adjustment, and neither familism nor interdependence served as moderators. Previous research has found that interdependent self-construal (i.e., adolescents defining themselves in terms of their relationships with their parents) moderates the relationship between the quality of parent–child relationship and emotional functioning among adolescents in both the United States and China (Pomerantz and Wang, 2009). Research among ethnic minority youth in the United States has shown that Latino American adolescents who value familism benefit more from parental control (e.g., monitoring) and had lower risks for substance use (Ramirez et al., 2004). It is possible that independence is a value that corresponds more to parental autonomy granting, and therefore serves as an influential moderator in our study. Future research should continue to examine the moderation effect of other cultural variables. Lastly, none of the cultural values at the classroom level predicted school functioning. It appears that between-individual differences, instead of between-classroom differences, in cultural values are more important for adolescents’ school functioning. Future research should consider other classroom-level predictors, such as attitudes toward learning and student–teacher relationships.

### Implications for Practice

Our results showed that parental autonomy granting benefits adolescents who value a strong sense of independence. Moreover, parental autonomy granting has an adverse effect for adolescents who value independence to a lesser degree. Therefore, educational psychologists, teachers, parent educators, and other helping professionals who are working with families can assess and assist parents to adjust their parenting practices based on the cultural values of their adolescents in order to optimize youth outcomes.

Furthermore, it is concerning that adolescents who reported higher levels of conflict with their parents reported more negative academic outcomes, which has been consistently documented in the literature. One possible implication is that schools or youth programs can develop curricula to teach parents and youth conflict resolution skills to help them learn appropriate ways to handle conflicts. These important life skills will not only help adolescents improve their family relationships but also may indirectly increase their academic performance. Parenting training may also help parents understand the challenges and stress adolescents experience while also teaching them ways to help their adolescents navigate these challenges.

### Limitations and Future Directions

This study contributes to current understandings of factors influencing Chinese adolescents’ school functioning. There are several limitations that need to be acknowledged. First, all data were based on adolescents’ self-report, and shared-method variance is of concern. Using adolescents’ perception of parenting practice may still produce useful results, because research has found that there were moderate but significant correlations between Chinese early adolescents’ and their mothers’ reports of parenting (Cheung et al., 2016). However, future studies should also collect data from parents and teachers to strengthen these findings. Second, the data were cross-sectional in nature. Studies utilizing longitudinal designs are needed to investigate the effects of these variables on youth outcomes over time. Third, in addition to autonomy support, future research should examine other parenting practices, such as psychological control, which has been found to interact with autonomy granting to predict adolescents’ outcomes (Kunz and Grych, 2013). Moreover, grade rank was a one-item measure of students’ last exam rank; therefore, it might not be a comprehensive assessment of students’ academic achievement. Future studies should collect students’ grades from school records or teachers’ reports to supplement grade ranking data. Finally, although not the focus of this study, it is important to mention that student–teacher relationships significantly contribute to school adjustment among adolescents (Longobardi et al., 2016). Future studies should examine how this variable may interact with cultural values to predict youth’s academic outcomes.

## Author Contributions

CWa developed the hypothesis, analyzed the data, and drafted the manuscript. KD analyzed the data and drafted the manuscript. LB and YX designed the study and collected the data. CWu assisted with data analyses. All authors read and approved the final manuscript.

## Conflict of Interest Statement

The authors declare that the research was conducted in the absence of any commercial or financial relationships that could be construed as a potential conflict of interest.
